# Changes in socioeconomic differences in fruit and vegetable consumption among statutorily retiring women: A longitudinal cohort study

**DOI:** 10.1016/j.jnha.2024.100425

**Published:** 2024-12-05

**Authors:** Anni Karjala, Jatta Salmela, Elina Mauramo, Aino Salonsalmi, Ossi Rahkonen, Tea Lallukka

**Affiliations:** Department of Public Health, University of Helsinki, P.O. Box 20, (Tukholmankatu 8 2B), 00014 Helsinki, Finland

**Keywords:** Socioeconomic disparities, Longitudinal study, Ageing, Nutrition, Population health

## Abstract

**Objectives:**

Socioeconomic differences in fruit and vegetable (F&V) consumption are recognized, but little is known about how these differences develop when moving from mid-life to older adulthood. We investigated the longitudinal changes in socioeconomic differences in F&V consumption in an ageing Finnish cohort, using occupational class as a measure of socioeconomic position. Additionally, we examined whether socioeconomic differences in F&V consumption changed over retirement transition.

**Design:**

An observational longitudinal cohort study with a 15–17-year follow-up.

**Setting and participants:**

The survey data used in this study were collected in four phases: 2000-02, 2007, 2012 and 2017. We included 2719 women who were 40–60-year-old in Phase 1. All participants transitioned to statutory retirement during the follow-up.

**Measurements:**

F&V consumption was measured in each phase as a part of a food frequency questionnaire (FFQ) and determined by the number of F&V consumption times per 4 weeks. We used linear mixed modeling for the analyses, and used age, marital status, education, income and BMI as covariates.

**Results:**

We found marked differences between occupational classes in F&V consumption. Semi-professionals used F&V most often and manual workers least often. In Phase 1, semi-professionals had 14.5 consumption times more per 4 weeks than manual workers, which is equivalent to ca. 0.5 daily consumption times. Differences between occupational classes showed a transient decrease in the beginning of the follow-up, followed by an increasing trend towards the last study phase. However, these changes were modest and overall differences between occupational classes changed only little over the follow-up period. Retirement did not markedly contribute to occupational class differences in F&V consumption.

**Conclusions:**

Our findings suggest that the socioeconomic differences in F&V consumption seen in mid-life persist in older adulthood and over retirement transition. The results imply that means to reduce socioeconomic differences in F&V use should be actively sought to support healthy ageing and reduce socioeconomic health differences in ageing populations. Workplace could be a fruitful ground for targeting these interventions.

## 1 Introduction

The population is ageing rapidly in high income countries, and the prevalence of chronic diseases is increasing [1,2]. Adequate fruit and vegetable (F&V) consumption is a cornerstone of healthy ageing. It is associated with a lower risk of key chronic diseases, with the benefits of F&V consumption being dose-dependent and cumulative over time [[3], [4], [5]]. A diet rich in F&V supports healthy ageing by, for example, slowing the rate of cognitive decline, preventing frailty, and improving physical function [[6], [7], [8], [9], [10]]. Diet rich in F&V has also been associated with a better quality of life in older age [11]. Despite older adults consuming F&V more often than younger adults and women consuming F&V more often than men, less than half of women who are 65 or older use fresh vegetables daily, and there has been a decreasing trend in these numbers in recent years [10,12].

F&V consumption is socioeconomically patterned, with individuals in low socioeconomic positions consuming F&V less often than those in higher socioeconomic positions [[13], [14], [15], [16], [17], [18]]. However, only few studies have looked at how these differences develop as individuals move into older adulthood. Most of the studies so far have been cross-sectional [15,18] or looked at associations of socioeconomic position to certain trajectories or changes in F&V consumption, rather than investigated the development of socioeconomic differences in F&V consumption [[19], [20], [21]]. Although socioeconomic differences in F&V consumption are recognized, there are no longitudinal studies investigating changes in socioeconomic differences in F&V consumption with ageing.

Different socioeconomic indicators measure different aspects of socioeconomic position, and different measures of socioeconomic position associate differently with F&V consumption [[13], [14], [15], [16]]. Occupational class is a key measure of socioeconomic position, reflecting the physical and psychosocial working conditions and the social status of an individual. It also indirectly describes the material resources as income level in higher occupational classes tends to be higher [22]. Previous studies have found that occupational class and F&V consumption tend to associate more strongly than some other socioeconomic indicators [21,24].

In the ageing population, the transition from employment to statutory retirement is a significant life event and known to influence health-related behaviours including diet [23]. However, changes in F&V consumption with retirement have been rarely examined in longitudinal settings [[24], [25], [26], [27], [28], [29]]. Furthermore, there is only little evidence on how socioeconomic position associates with this change [[24], [25], [26]].

To address the knowledge gap in how socioeconomic differences in F&V consumption change with ageing, we examined 1) whether occupational class differences in F&V consumption change over a 15–17-year follow-up time in a cohort of ageing female municipal employees and 2) if the transition to statutory retirement contributes to these changes.

## 2 Materials and methods

### 2.1 Study population

The Helsinki Health Study (HHS) survey data were used for the analyses. The Phase 1 data were collected in 2000−2002 among 40-, 45-, 50-, 55- and 60-year-old employees of the City of Helsinki, Finland. The number of respondents in Phase 1 was 8960 (response rate 67%). Follow-up surveys were conducted in 2007 (response rate 82%), 2012 (78%) and 2017 (83%) among all Phase 1 respondents. In our questionnaires, gender was derived from the questionnaires and thus subjective but defined in binary terms (“what is your gender: female/male”). A total of 3247 female participants transitioned to statutory retirement during the follow-up. We chose to include only women in our analyses, as the HHS cohort used in this study consists mainly (80%) of women, and the groups of men were small. Thus, more detailed analyses for men were not feasible.

Among the cohort, there were also individuals who first retired due to disability, and their retirement transformed into statutory retirement during the follow-up while ageing. Those individuals were not included in the analyses, as our focus was on the transition from working life to statutory retirement. Furthermore, some participants were absent from work during the follow-up before transitioning to statutory retirement due to other reasons (e.g., due to being a caregiver). Similarly, these participants were excluded from the analyses. Of those transitioning from work-life into statutory retirement, some of the participants returned to work after first retiring (*n* = 385). These participants were not included in our analyses, as the possible lifestyle changes these individuals have before and after retirement may be different compared to those who transitioned to statutory retirement. Those lacking information on occupational class (*n* = 51, 1.8%), education (*n* = 26, 0.9%), or body mass index (BMI, *n* = 24, 0.9%) at Phase 1 were excluded from the analyses. We also excluded respondents lacking data on F&V consumption at all four phases (*n* = 22, 0.2%) but included those who had responded to the F&V consumption on at least one of the survey phases. None of the participants lacked data on household income or marital status at all four phases. After these exclusions, 2719 respondents were included in the analyses.

### 2.2 Measuring fruit and vegetable consumption

The F&V consumption was measured by using a 22-item food frequency questionnaire (FFQ). The 2017 questionnaire included only 19 items for FFQ, but the content regarding F&V consumption remained essentially unchanged. In the FFQ, participants were asked to estimate how often they had consumed certain food items during the past 4 weeks. The items considered in this study were ‘fruit and berries’ and ‘fresh vegetables, root vegetables, and salads’. The response categories were: 1) not during the past 4 weeks, 2) 1–3 times a month, 3) once a week, 4) 2–4 times a week, 5) 5–6 times a week, 6) once a day and 7) twice or more daily. We calculated the number of consumption times per 4 weeks from these frequencies, by multiplying the frequency per day by 28. The resulting categories for consumption times per 4 weeks were thus 0, 2, 4, 12, 22, 28 and 56 for each item, and the maximum of monthly consumption times was 112 or more (accounting for 4 or more times a day). The conversion was done following previous procedures [21]. A total number of F&V consumption times per 4 weeks was calculated by adding up the consumption of the different response categories (‘fruit and berries’ and ‘fresh vegetables, root vegetables, salads’).

### 2.3 Occupational class

We divided participants into four occupational classes. The highest class included managers and professionals (e.g. teachers and doctors), the second highest semi-professionals (e.g. nurses), the second lowest routine non-manual employees (e.g. clerical work) and the lowest manual workers (e.g. transportation), following previous procedures [30,31]. We used the Phase 1 occupational class since our previous analyses have showen that there were only little transitions in occupational class during the follow-up [32]. The data were derived from the personnel register of the City of Helsinki among participants consenting to such a data linkage (78%) and completed from the questionnaires among the rest.

### 2.4 Statutory retirement

The data regarding retirement was obtained from the questionnaires (see section A.1 in appendix for specific questions). Those who stated their status as “full-time statutorily retired” were considered as statutorily retired at each study phase (except for Phase 1 where all respondents were employed). To investigate how F&V consumption changed with retirement transition, we created a time variable representing time in relation to retirement. The time of the first response on retirement was set as 0, and thus, the retirement occurred in between -1 and 0 (between two follow-up phases, 5 to 7 years apart), as no specific date for retirement was included in our dataset. As there were four phases, the timeline was between −3 and +2 depicting the time points in relation to retirement. As all individuals were employed in Phase 1, the respondents had a maximum of 3 response phases before and 3 phases after transitioning to retirement. The retired participants were placed on this timeline, each having a varying number of responses before and after retirement depending on when they retired. Of the participants, 44.1% (*n* = 1199) had responded at 3 phases before and one after retirement, 31.4% (*n* = 855) had had responded at two phases before and two after retirement, and 24.5% (*n* = 665) had responded at one phase before and three phases after retirement.

### 2.5 Covariates

We included age, marital status, household income, education and BMI as the covariates, based on previous research [13,21,24]. We included income and marital status as time-varying variables derived from the four phases. With the other covariates, values from Phase 1 were used. Age and BMI were included as continuous variables, and the other covariates as categorical. Marital status was categorized as married/cohabiting and single/divorced/widowed. The monthly income was divided by household size and weighted according to the modified Organisation for Economic Co-operation and Development equivalence scale [33]. Each respondent received the value of 1.0, other adults 0.5 and children 0.3. The respondents were divided into four hierarchical income groups, each of them consisting of approximately a quarter of the study population, following previous procedures [21]. Education was divided into three levels: higher (university degree), intermediate (matriculation or college examination) and basic (secondary or vocational school). Household income was measured by the total typical monthly household income. BMI was calculated from self-reported weight and height (kg/m^2^). We defined normal weight as BMI 18.5–24.9 kg/m^2^, overweight as BMI 25.0–29.9 kg/m^2^, and obesity as BMI ≥ 30 kg/m^2^, according to the World Health Organization’s BMI classification [34].

### 2.6 Statistical analyses

First, we calculated descriptive data on the main variables and covariates among the participants. Second, to estimate the change in F&V consumption in the occupational classes, we used linear mixed modeling (LMM). LMM is an extension of the general linear model, which specifies both fixed and random effects [35]. We chose this method to investigate longitudinal changes and to account for correlation by repeated measures from the same respondents. In addition, LMM allows the specification of both fixed and random effects, which was suitable for our purposes as participants were likely to have both different levels in F&V consumption and a different rate of change. In our analyses, we used maximum likelihood estimation for parameter estimation, and an autoregressive covariance structure. The residuals in our data were normally distributed, and model fit was evaluated using Akaike’s information criteria. All analyses were conducted using IBM SPSS (v.28.0).

To examine the development of F&V consumption in the occupational classes, we fitted a model with F&V consumption times per 4 weeks as the dependent variable, and occupational class, study phase and a two-variable interaction between occupational class and study phase as the independent variables. Similarly, to investigate the contribution of retirement to the change in F&V consumption, we fitted a model with an interaction of the time variable, indicating time in relation to retirement with F&V consumption times per 4 weeks, and a two-variable interaction between occupational class and the retirement time variable. Covariates were included as fixed effects in both models, and subject specific intercept and slope were included as random effects.

## 3 Results

Of the whole study population, 65% consumed F&V over 56 times per 4 weeks in Phase 1 (Table 1). Averaging this over 4 weeks makes twice per day. Of the occupational classes, semi-professionals consumed F&V most often, with two thirds (74.9%) of the class consuming F&V over 56 times per 4 weeks (more than twice per day). Most of the study population were married or cohabiting (67.9%), and half were normal weight (50.6%). Half (49.5%) had matriculation or college examination as an educational background.

**Table 1:** Characteristics of the respondents by occupational class in Phase 1 (2000–2002).

	All participants	Professionals	Semi-professionals	Non-manual employees	Manual workers	*P-*value*
	*n* = 2719	*n* = 876	*n* = 439	*n* = 1119	*n* = 285	
F&V consumption times per 4 weeks, % (n)						p < 0.001
≤ 56 (less than twice per day)	35.1% (946)	31.7% (277)	25.1% (109)	38.6% (427)	47.3% (133)	
>56 (twice or more daily)	64.9%, (1749)	68.3%, (596)	74.9% (326)	61.4% (679)	52.7% (148)	
Age						p = 0.02
40–49	4.5% (123)	6.7% (59)	3.2% (14)	3.5% (39)	3.9% (11)	
50−60	95.5% (2596)	93.3% (817)	96.8% (425)	96.5% (1080)	96.1% (274)	
Body mass index (BMI), % (n)						p < 0.001
Obese (BMI ≥ 30)	14.1% (379)	11.9% (103)	12.5% (54)	15.0% (166)	19.8% (56)	
Overweight (BMI 25.0–29.9)	34.5% (927)	29.0% (251)	35.1% (152)	36.9% (408)	.41.0% (116)	
Normal weight (BMI ≤ .24.9)	50.8% (1382)	58.4% (512)	51.7% (227)	47.5% (532)	39.0% (111)	
Marital status, % (n)						p = 0.02
Widowed, divorced or single	32.1% (872)	28.6% (250)	32.0% (140)	33.6% (375)	37.7% (107)	
Married or cohabiting	67.9% (1841)	71.4% (625)	68.0% (298)	66.4% (741)	62.3% (177)	
Education, % (n)						p < 0.001
Secondary or vocational school	22.8% (621)	0.9% (8)	11.6% (51)	36.6% (410)	53.3% (152)	
Matriculation or college examination	49.6% (1348)	21.7% (190)	78.8% (346)	60.9% (681)	46.0% (131)	
University degree	27.6%(750)	77.4% (678)	9.6% (42)	2.5% (28)	0.7% (2)	
Household income, % (n)						p < 0.001
Lowest quartile	19.7% (521)	5.3% (46)	15.1% (65)	28.5% (307)	37.5% (103)	
Second lowest quartile	22.0% (581)	14.3% (123)	21.1 %( 91)	26.5% (285)	30.1% (82)	
3rd lowest quartile	23.0% (606)	25.9% (223)	23.2% (100)	22.0% (237)	16.9% (46)	
Highest quartile	35.3% (932)	54.4% (468)	40.6% (175)	23.0% (248)	15.1% (41)	

* P-values were calculated using the chi-square test of independence.

**Fig. 1:**
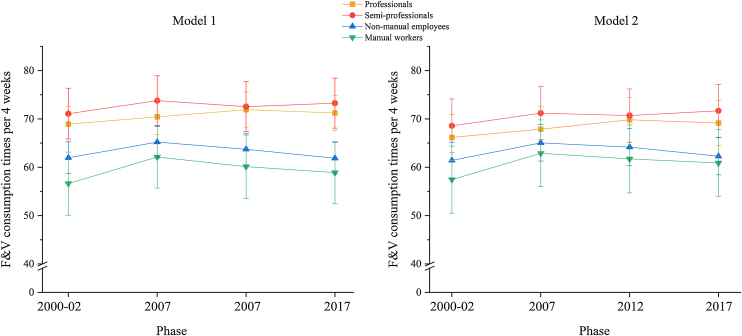
Model estimated marginal means and their 95% confidence intervals of fruit and vegetable (F&V) consumption times per 4 weeks in occupational classes, at Phases 1–4 (*n* = 2719). The left-side graph is for model 1 (unadjusted) and the right-side graph is for model 2 (adjusted for age, marital status, household income, education, and BMI).

Over the follow-up, semi-professionals had highest F&V consumption in all study phases, followed by professionals, non-manual employees, and manual workers (Fig. 1). The difference between the class with highest (semi-professionals) and the two classes with lowest F&V consumption (non-manual employees and manual workers) over the follow-up was statistically significant and persisted after adjusting for covariates (p < 0.001). In Phase 1, the difference in F&V consumption between semi-professionals and manual workers was 14.5 times per 4 weeks in the unadjusted model and 11.1 when adjusting for covariates (age, marital status, household income, education, and BMI). The respective difference in F&V consumption between semi-professionals and non-manual employees in Phase 1 were 9.1 (unadjusted) and 7.1 (adjusted model) consumption times per 4 weeks (Fig. 1).

During the follow-up, all occupational classes slightly increased their F&V consumption from Phase 1 to 4, except for non-manual employees for whom the F&V consumption did not change (≤1 consumption time per 4 weeks from Phase 1 to 4 (Fig. 1 and Table A.2)). In all classes, there was an increase in F&V consumption between phases 1 and 2, most prominently for manual workers and non-manual employees. This change resulted in transient narrowing in occupational class differences in Phase 2, after which there was a steeper decline in F&V consumption for non-manual employees and manual workers (Fig. 1). The difference between semi-professionals and manual workers at the last phase did not change from Phase 1 to Phase 4 (≤1 consumption time per 4 weeks, in both unadjusted and adjusted models (Fig. 1, Table A.2 and A.3)), but the difference between semi-professionals and non-manual employees increased from 9.1 to 14.4 consumption times per 4 weeks from Phase 1 to 4 (Fig. 1, Table A.2). After adjusting for covariates, this increase was from 7.2 to 9.4 consumption times per 4 weeks (Table A.3). Despite the increasing trend in the last follow-up phase, the overall change in occupational class differences over the follow-up time was not statistically significant (interaction for occupational class*phase p = 0.66, Table A.6).

**Fig. 2:**
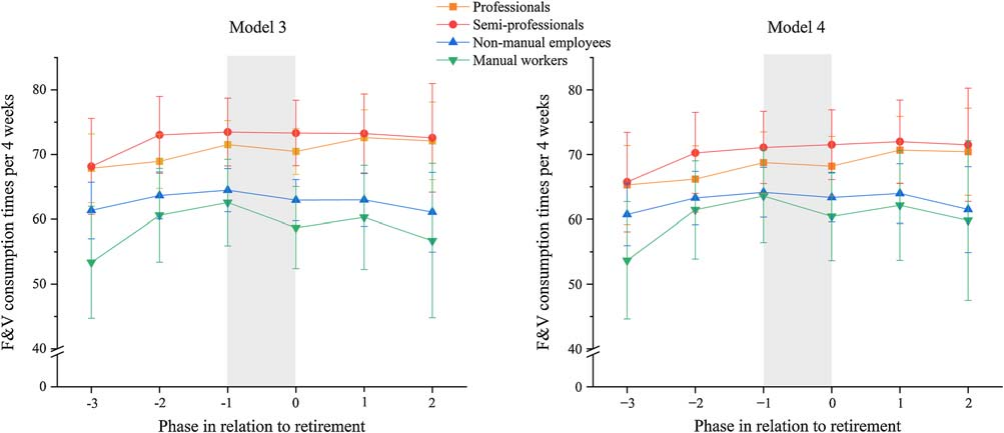
Model estimated marginal means their 95% confidence intervals of fruit and vegetable (F&V) consumption times per 4 weeks in occupational classes, in relation to the transition into statutory retirement (n = 2719). Retirement occurs between time points −1 and 0 (grey area). The left-side graph is for model 3 (unadjusted) and the right-side graph is for model 4 (adjusted for age, marital status, household income, education, and BMI).

During retirement transition, F&V consumption changed little in the occupational classes (Fig. 2). The largest change in F&V consumption was seen in the manual workers, for whom a decrease of 3.9 times per 4 weeks was seen with retirement transition (Fig. 2, Table A.4). Adjusting for covariates reduced this decline slightly, with the decrease being 3.1 times per 4 weeks in the adjusted model (Fig. 2, Table A.5). The non-manual employees also showed a small decrease of 1.5 times per 4 weeks in their F&V consumption with retirement, but this change was diminished to 0.8 when adjusting for covariates (Fig. 2, Table A.4 and A.5). With semi-professionals and professionals, the F&V consumption per 4 weeks remained essentially unchanged (≤1 consumption time per 4 weeks) with retirement transition. However, these changes in occupational class differences over the retirement transition were not statistically significant.

## 4 Discussion

We investigated changes in socioeconomic differences in F&V consumption in a cohort of ageing women moving from mid-life into older adulthood, using occupational class as the measure for socioeconomic position. We also examined whether transition from working life to statutory retirement contributed to these changes. We found that differences between occupational class with highest F&V consumption (semi-professionals) and the two classes with lowest F&V consumption (manual workers and non-manual employees) were marked and remained statistically significant throughout the follow-up period of 15–17 years. The difference between the group with highest (semi-professionals) and lowest F&V consumption (manual workers) was 14.5 consumption times per 4 weeks in Phase 1, which is equivalent to ca. 0.5 daily consumption times. There was a transient decrease in occupational class differences in the beginning of the follow-up, followed by an increasing trend towards the end of the follow-up. Furthermore, transitioning to statutory retirement did not significantly change the occupational class differences in F&V consumption. Our results suggest that the socioeconomic differences in F&V consumption observed in mid-life persist in older adulthood among retiring women. More research with longer follow-up time is warranted to confirm the increasing trend seen in these differences at the end of follow-up in our study.

### 4.1 Comparison with previous studies

There are no previous longitudinal studies investigating the change of socioeconomic differences in F&V consumption with ageing. Some studies with a focus on wider dietary measures have found contradicting results on the development of socioeconomic differences. A recent study from Brazil reported a decrease in socioeconomic differences in consumption of plant-based and minimally processed foods over a 10-year follow-up [36], which is contradictory to our findings. However, the study did not investigate F&V consumption but wider dietary measures, included participants with varying ages (over 20 years of age) and used education and income as socioeconomic indicators. Our previous study, conducted with the Helsinki Health study cohort, found that occupational class differences in food consumption changed little over a 5–7-year follow-up among younger (40–67 years of age) male and female participants, with an increase in F&V use seen for all occupational classes [37]. Previous evidence suggests that dietary habits and socioeconomic differences in nutrition, formed early in the life-course, remain even in older adulthood [38]. Our results support this evidence, suggesting that the socioeconomic differences in F&V consumption seen in mid-life persist into older adulthood.

Socioeconomic differences in F&V consumption remained large over the follow-up time. Overall, the participants in our study consumed F&V less frequently than what is generally recommended. Only two thirds of the whole study population reported using F&V more than 56 times per 4 weeks (or more than twice a day) in Phase 1. The manual workers class had the lowest F&V consumption, with only half of the group reporting F&V consumption over 56 times per 4 weeks (or more than twice a day). The size of the difference in F&V consumption (over 14 portions per 4 weeks) between semi-professionals and manual workers is large, considering the readily rare F&V consumption, and the recommended intake in Finland being 500 g or 5–6 portions of F&V per day [12], and by WHO being 400 g or 5 portions per day [39]. F&V consumption is known to be the key mediator of health benefits of healthy diet in a dose-dependent and cumulative manner. Furthermore, diet quality contributes to socioeconomic differences in, for example, obesity and cardiovascular morbidity and mortality [40,41]. As the socioeconomic differences in F&V consumption remained large over the long 15–17-year follow-up in our study, those in low socioeconomic position are less likely to gain the long-term benefits of F&V consumption, suggesting further accumulation of health disadvantage in groups that are readily more vulnerable.

There was a transient decrease in socioeconomic differences between Phases 1 (in 2000–02) and 2 (in 2007). This finding is in line with previous reports from Finland [37,42], where an increase in F&V consumption has been observed during this period. After this, the differences between occupational classes showed a modestly increasing trend towards the last study phase. This observation could have been, for instance, due to economic aspects, as the Finnish and global economy experienced drastic changes with the recession in 2008. This could have impacted the greater reduction in F&V consumption for manual and non-manual employees in Phases 3 and 4 given that those in lower occupational classes tend to be financially more vulnerable, and the established association of income with changes in F&V consumption [21]. However, the observed trend remained even after adjusting for household income, so the transient decrease and the following increase in socioeconomic differences in F&V consumption likely have other explanations, such as changes in food culture and public health campaigns or policies. After Phase 2, there was an increasing trend between occupational classes in F&V consumption, especially between semi-professionals and non-manual employees. However, this change was only modest, and more research with longer follow-up time is warranted to confirm if this trend continues as individuals age further.

We found that socioeconomic differences in F&V consumption did not markedly change with retirement transition. Previous studies have reported contradictory results for the change of F&V consumption with transition into statutory retirement. Some studies have found little change in F&V use with retirement among women [28], whereas others have reported increases in F&V consumption [43]. In our previous study, we found that higher socioeconomic position was associated with higher likelihood to adequately consume F&V after transitioning into statutory retirement [29]. The manual workers reduced their F&V consumption with retirement, and the decline continued after the retirement transition. The non-manual employees also showed a decrease in their F&V consumption during and after retirement, but this change was diminished when adjusting for age, marital status, education, household income and BMI. With the professionals and semi-professionals, little change in F&V consumption was observed with retirement. Although the decline seen in the group of manual workers was small and did not significantly contribute to the observed differences, this implies that those belonging to low occupational class may be more sensitive to reducing their F&V consumption with retirement than the other groups. Furthermore, our findings suggest that the socioeconomic differences in F&V consumption seemed to show a modestly increasing pattern after the retirement transition. However, the group of manual workers was small and the confidence intervals rather wide, which weakens the consistency of these results. It is possible that the socioeconomic differences in F&V consumption increase in later life, and further studies on the contribution of retirement transition are needed.

We chose to use occupational class as the measure of socioeconomic position, as our cohort was a municipal one, and occupational class is known to associate with F&V consumption more strongly than some other socioeconomic indicators [21,44]. The difference seen in F&V consumption could thus be mediated through work-related factors such as high work strain, low job control and long or irregular working hours, which are reflected by occupational class [17,45]. These working conditions could, for example, limit the possibility for a regular eating pattern or preparing healthy, F&V rich meals. The atmosphere and culture in the working community can also shape the eating patterns in the occupational classes [46]. This atmosphere could also impact how those in different occupational classes reported their F&V consumption, as those in higher occupational class might feel more pressure towards eating healthy and thus be prone to overestimate their F&V consumption.

Adjusting for covariates, that is age, marital status, education, household income and BMI, slightly decreased the occupational class differences and their change over time. It is known that those with higher occupational class tend to have higher educational attainment and income levels [47]. Furthermore, education and income are known to impact eating behaviour [16,44], as those with higher educational level might have better health literacy whereas those with better income tend to have better material resources and thus financial access to adequate F&V consumption. These factors can thus indirectly contribute to the observed differences. However, adjusting for these covariates did not completely attenuate the occupational class differences. This implies that although socioeconomic indicators are overlapping, they still depict somewhat different aspects of socioeconomic differences in F&V consumption [13].

### 4.2 Strengths and limitations

Strengths of our study included a large dataset and a good response rate at baseline and follow-up points. Our data contained a long follow-up on the same cohort, and enabled investigating longitudinal changes in F&V consumption. The long follow-up also enabled us to estimate the contribution of retirement transition to these changes. However, as our cohort consisted of initially employed women in the municipal sector, the results cannot be directly applied to a general population or other population groups. We chose to include only women in our analyses, as the HHS cohort used in this study consists mainly (80%) of women, and the groups of men were small. As F&V consumption patterns and occupations of women are different to those of men [24], and more detailed subgroup analysis by gender, occupational class, and retirement status as well as inclusion of covariates were not feasible for men due to the small group size. Additionally, as municipal workers are mainly women in the City of Helsinki and in Finland in general, our cohort represents this population well.

Age, period and cohort effects are inherent in longitudinal studies and can all affect health behaviours differently [48]. Our study had a long follow-up period, and although adjusting the analyses for age and modelling the changes in F&V consumption in two different ways (i.e., over the study phases and over time in relation to retirement), we could not account for all the factors that could have changed in the surrounding environment or culture, possibly shaping the F&V consumption of the participants, or factors such as public health policies or societal change. These effects might have impacted socioeconomic differences in F&V consumption over the follow-up. Additionally, development socioeconomic differences in F&V consumption might differ between generations, and wider age distribution in our sample could have enabled us to consider cohort effects in these differences. The presence of these effects underlines the need for further studies with multiple cohorts and time periods, and with a specific focus on age, period, and cohort effects in the development of socioeconomic differences in F&V consumption.

In our dataset, F&V consumption was measured by asking the participants to evaluate the consumption frequency. The participants were thus susceptible for recalling bias, as some studies have reported that participants tend to overestimate their F&V consumption in questionnaires [49]. Additionally, our FFQ did not include the amount or quality of F&V that were consumed. The FFQ questions included in this study, for example, did not include cooked vegetables, and thus the total levels of F&V consumed is likely to be somewhat higher. Frequent F&V consumption does not necessarily guarantee an adequate F&V intake if the portion sizes are very small or if there is little variety in the F&Vs consumed. Furthermore, as the highest category of consumption was ‘two or more times daily’, higher F&V consumption, for instance 5 times per day or more, was not captured. Thus, our results do not paint a very precise picture of the participants’ F&V consumption. However, our aim was to investigate broader socioeconomic patterns in F&V consumption, where this approach has been proven useful [21,24,29,37].

We chose to investigate F&V consumption, as it is a key mediator of benefits of healthy nutrition. With ageing, however, other aspects of nutrition, such as adequate protein and energy intake are also essential [50]. Furthermore, other healthy dietary patterns, such as fish or wholegrain use, or unhealthy patterns, such as excessive use of red meat or saturated fats, were not examined in our study. The results do not thus provide the whole picture of participants’ diets. Our measure of F&V consumption also combined fruit, vegetable, and berry consumption. Previous research has shown that the change seen with retirement may be different for these F&V subcategories [29]. More research on the change in socioeconomic differences in fruit, vegetable and berry use as well as other aspects of healthy nutrition is thus needed.

In studies with follow-up questionnaires, non-response and attrition are inherently present. It is possible that our dataset contained selection bias towards those with better health and higher F&V consumption. Those with a heavier disease burden did not possibly respond to our questionnaire as often or were dropped out earlier. This would imply that the socioeconomic differences in F&V could be even larger than the ones reported in this study, as those in lower socioeconomic positions are known to have poorer health. It is also possible that there was attrition due to death. However, the response rate was high during the follow-up, and only a small population dropped out during the follow-up, which reduces the likelihood of biased results due to attrition.

In our dataset, the exact date of transitioning to retirement was not known. Thus, the retirement transition was described as time in relation to the first phase, when the participant had transitioned to retirement. As the time between phases was up to 5 years, the first response phase on retirement could have been up to 5 years before or after the actual transition. However, we consider that our method is adequately capable of capturing the long-term contributions of retirement transition on F&V consumption, which was our interest.

## 5 Conclusions

We found that socioeconomic differences in F&V consumption changed little over a 15–17-year follow-up time and remained substantial. There was a transient decrease in these differences between phases 1 and 2, followed by a modestly increasing trend towards the end of the follow-up. More research with longer follow-up time is needed to confirm the increasing trend seen at the last follow-up phase. Transitioning into statutory retirement contributed little to the observed differences. Our results support the evidence that socioeconomic differences in F&V consumption develop in earlier life and are persistent even through retirement transition. With healthy nutrition impacting both long-term development of chronic disease and being crucial for healthy ageing, these differences should be targeted effectively to reduce socioeconomic health disparities and improve health of ageing populations. Supporting eating at the workplace canteens could improve diet quality during workdays [51], and these healthy eating habits may remain into older adulthood and over the retirement transition. Furthermore, the large socioeconomic differences in F&V consumption and their stagnant nature during ageing should be targeted when planning society-level interventions such as price policies or taxation.

## CRediT authorship contribution statement

Anni Karjala: Conceptualization, methodology, investigation, writing – original draft preparation, visualization. Jatta Salmela: Conceptualization, methodology, writing – reviewing and editing. Elina Mauramo: Conceptualization, writing – reviewing and editing, Aino Salonsalmi Conceptualization, writing – reviewing and editing, Tea Lallukka: Conceptualization, writing – reviewing and editing, Ossi Rahkonen: Conceptualization, writing – reviewing and editing.

## Ethics declaration

All procedures performed in studies involving human participants were in accordance with the ethical standards of the institutional and national research committee and with the 1964 Helsinki declaration and its later amendments or comparable ethical standards and the Finnish law. The Helsinki Health Study protocol has been approved by ethics committees of the Department of Public Health of University of Helsinki, and the health authorities of City of Helsinki.

## Funding

JS and TL were supported by the Academy of Finland (Grant #330527), AK was supported by Juho Vainio Foundation (grant #202300131), OR was supported by the Juho Vainio Foundation (Grant #202300041) and the Ministry of Culture and Education, Finland (Grant #OKM/76/626/2023) and EM was supported by Juho Vainio Foundation (Grant #202400393). Open access funded by Helsinki University Library.

## Data availability

Data can be obtained through reasonable requests. The Helsinki Health Study survey data cannot be publicly released due to stringent data protection laws and regulations. Use of the data is restricted to scientific research and collaboration with the research group's partners, subject to a reasonable request and study plan. Further details regarding the availability of the survey data can be obtained by contacting the Helsinki Health Study research group at kttl-hhs@helsinki.fi.

## Declaration of competing interest

The authors declare that they have no conflict of interests.

The authors declare the following financial interests/personal relationships which may be considered as potential competing interests:

Anni Karjala reports financial support was provided by Juho Vainio Fundation. Jatta Salmela, Tea Lallukka reports financial support was provided by Research Council of Finland. Ossi Rahkonen, Elina Mauramo reports financial support was provided by Juho Vainio Fundation. Ossi Rahkonen reports financial support was provided by Finnish Ministry of Education and Culture. If there are other authors, they declare that they have no known competing financial interests or personal relationships that could have appeared to influence the work reported in this paper.

## Glossary

BMI: Body mass index
CI: Confidence interval
F&V: Fruit and vegetable
FFQ: Food frequency questionnaire
HHS: Helsinki Health Study
LMM: Linear mixed models
