# Supplementary material for: Changes in socioeconomic differences in fruit and vegetable consumption among statutorily retiring women: A longitudinal cohort study
Source: J Nutr Health Aging. 2024 Dec 5;29(1):100425. doi: 10.1016/j.jnha.2024.100425 (PMC12179988; doi:10.1016/j.jnha.2024.100425)
Supplement: Supplementary file 1 [file mmc1.docx]

Section A.1. Translation of retirement questions in the questionnaires. phases 2-4. Original questionnaires were in Finnish and Swedish.

**Which of the following best describes your current situation?**

- Full-time job
- Part-time job
- Part-time statutory retirement or part-time early statutory retirement
- Statutory retirement
- Over 6 months long sickness absence
- Full-time or part-time disability retirement
- Other

Table A2. Model estimated marginal means (model 1) and their upper and lower 95% confidence intervals (CIs) for fruit and vegetable (F&V) consumption in different occupational classes at study phases 1-4. for a cohort of women (n=2719) from the Helsinki Health study.

| **Phase** | | **Professionals** | | **CI (high)** | | **CI (low)** | | **Semi-professionals** | | **CI (high)** | | **CI (low)** | | **Non-manual employees** | | **CI (high)** | | **CI (low)** | | **Manual workers** | | **CI (high)** | | **CI (low)** | |
| --- | --- | --- | --- | --- | --- | --- | --- | --- | --- | --- | --- | --- | --- | --- | --- | --- | --- | --- | --- | --- | --- | --- | --- | --- | --- |
| 1 | | 68.9 | | 67.1 | | 70.8 | | 71.1 | | 68.5 | | 73.7 | | 62.0 | | 60.3 | | 63.6 | | 56.6 | | 53.4 | | 59.9 | |
| 2 | | 70.4 | | 68.6 | | 72.3 | | 73.8 | | 71.2 | | 76.4 | | 65.2 | | 63.6 | | 66.8 | | 62.1 | | 58.9 | | 65.3 | |
| 3 | | 71.9 | | 70.1 | | 73.7 | | 72.5 | | 70.0 | | 75.1 | | 63.7 | | 62.1 | | 65.4 | | 60.1 | | 56.9 | | 63.4 | |
| 4 | | 71.3 | | 69.4 | | 73.1 | | 73.3 | | 70.7 | | 75.9 | | 61.9 | | 60.2 | | 63.5 | | 58.9 | | 55.7 | | 62.1 | |

Table A.3. Model estimated marginal means (model 2) and their upper and lower 95% confidence intervals (CIs) for fruit and vegetable (F&V) consumption in different occupational classes at study phases 1-4. for a cohort of women (n=2719) from the Helsinki Health study. Adjusted for age. marital status. education. income and BMI.

| **Phase** | **Professionals** | **CI (high)** | **CI (low)** | **Semi-professionals** | **CI (high)** | **CI (low)** | **Non-manual employees** | **CI (high)** | **CI (low)** | **Manual workers** | **CI (high)** | **CI (low)** |
| --- | --- | --- | --- | --- | --- | --- | --- | --- | --- | --- | --- | --- |
| 1 | 66.2 | 63.8 | 68.6 | 68.6 | 65.8 | 71.4 | 61.4 | 59.5 | 63.3 | 57.4 | 54.0 | 60.9 |
| 2 | 67.9 | 65.5 | 70.2 | 71.2 | 68.5 | 74.0 | 65.1 | 63.2 | 66.9 | 62.9 | 59.4 | 66.4 |
| 3 | 69.8 | 67.5 | 72.2 | 70.7 | 68.0 | 73.5 | 64.2 | 62.3 | 66.1 | 61.7 | 58.2 | 65.2 |
| 4 | 69.2 | 66.8 | 71.5 | 71.7 | 69.0 | 74.4 | 62.3 | 60.4 | 64.2 | 60.9 | 57.5 | 64.3 |

Table A.4. Model estimated marginal means (model 3) and their upper and lower 95% confidence intervals (CIs) for fruit and vegetable (F&V) consumption in different occupational classes at phases in relation to retirement. for a cohort of women (n=2719) from the Helsinki Health study. Retirement occurs between phases -1 to 0 in relation to retirement.

| **Phase in relation to retirement** | **Professionals** | **CI (high)** | **CI (low)** | **Semi-professionals** | **CI (high)** | **CI (low)** | **Non-manual employees** | **CI (high)** | **CI (low)** | **Manual workers** | **CI (high)** | **CI (low)** |
| --- | --- | --- | --- | --- | --- | --- | --- | --- | --- | --- | --- | --- |
| -3 | 67.9 | 65.2 | 70.5 | 68.1 | 64.4 | 71.9 | 61.4 | 59.2 | 63.5 | 53.4 | 49.0 | 57.7 |
| -2 | 68.9 | 66.8 | 71.0 | 73.0 | 70.0 | 76.0 | 63.7 | 61.8 | 65.5 | 60.6 | 57.0 | 64.2 |
| **-1** | **71.5** | **69.7** | **73.4** | **73.5** | **70.8** | **76.1** | **64.5** | **62.8** | **66.1** | **62.6** | **59.2** | **65.9** |
| **0** | **70.5** | **68.7** | **72.2** | **73.3** | **70.8** | **75.8** | **62.9** | **61.3** | **64.5** | **58.7** | **55.6** | **61.9** |
| 1 | 72.6 | 70.5 | 74.7 | 73.2 | 70.2 | 76.3 | 63.0 | 61.0 | 65.0 | 60.3 | 56.3 | 64.3 |
| 2 | 72.1 | 69.1 | 75.1 | 72.6 | 68.4 | 76.8 | 61.1 | 58.0 | 64.2 | 56.7 | 50.7 | 62.7 |

Table A.5. Model estimated marginal means (model 4) and their upper and lower 95% confidence intervals (CIs) for fruit and vegetable (F&V) consumption in different occupational classes at phases in relation to retirement. for a cohort of women (n=2719) from the Helsinki Health study. Retirement occurs between phases -1 to 0 in relation to retirement. Adjusted for age. marital status. education. income and BMI.

| **Phase in relation to retirement** | **Professionals** | **CI (high)** | **CI (low)** | **Semi-professionals** | **CI (high)** | **CI (low)** | **Non-manual employees** | **CI (high)** | **CI (low)** | **Manual workers** | **CI (high)** | **CI (low)** |
| --- | --- | --- | --- | --- | --- | --- | --- | --- | --- | --- | --- | --- |
| -3 | 65.3 | 62.2 | 68.3 | 65.7 | 61.9 | 69.6 | 60.7 | 58.3 | 63.1 | 53.7 | 49.1 | 58.2 |
| -2 | 66.2 | 63.6 | 68.8 | 70.2 | 67.1 | 73.4 | 63.3 | 61.2 | 65.3 | 61.4 | 57.7 | 65.2 |
| **-1** | **68.7** | **66.4** | **71.1** | **71.1** | **68.3** | **73.9** | **64.1** | **62.2** | **66.1** | **63.6** | **60.0** | **67.1** |
| **0** | **68.2** | **65.9** | **70.5** | **71.5** | **68.8** | **74.2** | **63.4** | **61.5** | **65.2** | **60.4** | **57.0** | **63.8** |
| 1 | 70.7 | 68.1 | 73.3 | 72.0 | 68.8 | 75.2 | 64.0 | 61.7 | 66.3 | 62.1 | 57.9 | 66.4 |
| 2 | 70.4 | 67.1 | 73.8 | 71.5 | 67.1 | 75.9 | 61.5 | 58.2 | 64.8 | 59.8 | 53.6 | 66.0 |

Table A.6. Fixed effect estimates for models 1 and 2. for the change in occupational class differences over the follow-up. Model 1 was unadjusted. Model 2 adjusted for age. marital status. education. income and BMI.

| Fixed Effects | Model 1 Coefficient | Model 1 Std. Error | Model 1 t-value | Model 1 p-value | Model 2 Coefficient | Model 2 Std. Error | Model 2 t-value | Model 2 p-value |
| --- | --- | --- | --- | --- | --- | --- | --- | --- |
| Intercept | 71.3 | 0.9 | 76.2 | <0.001 | 76.3 | 5.9 | 12.9 | <0.001 |
| Manual workers | -12.3 | 1.9 | -6.5 | <0.001 | -8.3 | 2.3 | -3.6 | <0.001 |
| Non-manual employees | -9.4 | 1.2 | -7.5 | <0.001 | -6.9 | 1.7 | -4.0 | <0.001 |
| Semi-professionals | 2.0 | 1.6 | 1.2 | 0.21 | 2.5 | 1.9 | 1.3 | 0.18 |
| Professionals | Ref. | . | . | . | Ref. | . | . | . |
| Phase 1 (2000-02) | -2.3 | 1.0 | -2.4 | 0.017 | -3.0 | 1.0 | -3.0 | 0.003 |
| Phase 2 (2007) | -0.8 | 1.0 | -0.8 | 0.4 | -1.3 | 1.0 | -1.3 | 0.2 |
| Phase 3 (2012) | 0.7 | 0.9 | 0.7 | 0.48 | 0.7 | 1.0 | 0.7 | 0.50 |
| Phase 4 (2017) | Ref. | . | . | . | Ref. | . | . | . |
| Age | - | - | - | - | 0.1 | 0.1 | 0.5 | 0.59 |
| BMI | - | - | - | - | -0.3 | 0.1 | -2.5 | 0.012 |
| Marital status |  |  |  |  |  |  |  |  |
| Widowed. divorced or single | - | - | - | - | -1.8 | 0.9 | -2.0 | 0.051 |
| Married or cohabiting | - | - | - | - | Ref. | . | . | . |
| Education |  |  |  |  |  |  |  |  |
| Secondary or vocational school | - | - | - | - | -3.3 | 1.8 | -1.9 | 0.06 |
| Matriculation or college examination | - | - | - | - | 1.9 | 1.5 | 1.3 | 0.2 |
| University degree | - | - | - | - | Ref. | . | . | . |
| Income |  |  |  |  |  |  |  |  |
| Lowest quartile | - | - | - | - | -4.5 | 0.9 | -5.2 | <0.001 |
| Second lowest quartile | - | - | - | - | -2.9 | 0.8 | -3.8 | <0.001 |
| 3rd lowest quartile | - | - | - | - | -1.9 | 0.7 | -2.8 | 0.005 |
| Highest quartile | - | - | - | - | Ref. | . | . | . |
| Interaction Terms |  |  |  |  |  |  |  |  |
| Manual workers * Phase 1 | 0.0 | 2.0 | 0.0 | 0.99 | -0.5 | 2.0 | -0.2 | 0.8 |
| Manual workers * Phase 2 | 4.0 | 2.0 | 2.0 | 0.04 | 3.3 | 2.0 | 1.6 | 0.11 |
| Manual workers * Phase 3 | 0.5 | 2.0 | 0.3 | 0.78 | 0.2 | 2.0 | 0.1 | 0.93 |
| Manual workers * Phase 4 | Ref. | . | . | . | Ref. | . | . | . |
| Non-manual employees * Phase 1 | 2.4 | 1.3 | 1.9 | 0.06 | 2.1 | 1.3 | 1.6 | 0.11 |
| Non-manual employees * Phase 2 | 4.2 | 1.3 | 3.2 | 0.002 | 4.0 | 1.3 | 3.0 | 0.002 |
| Non-manual employees * Phase 3 | 1.2 | 1.3 | 0.9 | 0.35 | 1.2 | 1.3 | 0.9 | 0.35 |
| Non-manual employees * Phase 4 | Ref. | . | . | . | Ref. | . | . | . |
| Semi-professionals * Phase 1 | 0.1 | 1.7 | 0.1 | 0.93 | -0.2 | 1.7 | -0.1 | 0.93 |
| Semi-professionals * Phase 2 | 1.3 | 1.7 | 0.8 | 0.43 | 0.8 | 1.7 | 0.5 | 0.65 |
| Semi-professionals * Phase 3 | -1.4 | 1.6 | -0.8 | 0.4 | -1.7 | 1.7 | -1.0 | 0.32 |
| Semi-professionals * Phase 4 | Ref. | . | . | . | Ref. | . | . | . |
| Professionals * Phase 1 | Ref. | . | . | . | Ref. | . | . | . |
| Professionals * Phase 2 | Ref. | . | . | . | Ref. | . | . | . |
| Professionals * Phase 3 | Ref. | . | . | . | Ref. | . | . | . |
| Professionals * Phase 4 | Ref. | . | . | . | Ref. | . | . | . |

Ref. indicates reference category

Phase in rel. to retirement indicates phase in relation to retirement

Table A.7. Fixed effect estimates for models 3 and 4. for the change in occupational class differences with transition into retirement. Retirement occurs between phases -1 to 0 in relation to retirement. Model 3 was unadjusted. Model 4 adjusted for age. marital status. education. income and BMI.

| Fixed Effects | Model 3 Coefficient | Model 3 Std. Error | Model 3 t-value | Model 3 p-value | Model 4 Coefficient | Model 4 Std. Error | Model 4 t-value | Model 4 p-value |
| --- | --- | --- | --- | --- | --- | --- | --- | --- |
| Intercept | 72.1 | 1.5 | 47.0 | <0.001 | 84.0 | 6.4 | 13.1 | <0.001 |
| Manual workers | -15.4 | 3.4 | -4.5 | <0.001 | -10.6 | 3.7 | -2.9 | 0.004 |
| Non-manual employees | -11.0 | 2.2 | -5.0 | <0.001 | -8.9 | 2.5 | -3.6 | <0.001 |
| Semi-professionals | 0.5 | 2.6 | 0.2 | 0.86 | 1.1 | 2.8 | 0.4 | 0.71 |
| Professionals | Ref. | . | . | . | Ref. | . | . | . |
| Phase in rel. to retirement -3 | -4.2 | 1.9 | -2.2 | 0.03 | -5.1 | 1.9 | -2.6 | 0.008 |
| Phase in rel. to retirement -2 | -3.2 | 1.7 | -1.8 | 0.07 | -4.2 | 1.7 | -2.4 | 0.02 |
| Phase in rel. to retirement -1 | -0.6 | 1.6 | -0.4 | 0.72 | -1.7 | 1.6 | -1.1 | 0.29 |
| Phase in rel. to retirement 0 | -1.6 | 1.6 | -1.0 | 0.3 | -2.2 | 1.6 | -1.4 | 0.16 |
| Phase in rel. to retirement 1 | 0.5 | 1.6 | 0.3 | 0.8 | 0.3 | 1.6 | 0.2 | 0.88 |
| Phase in rel. to retirement 2 | Ref. | . | . | . | Ref. | . | . | . |
| Age | - | - | - | - | -0.1 | 0.1 | -0.6 | 0.55 |
| BMI | - | - | - | - | -0.3 | 0.1 | -2.5 | 0.01 |
| Marital status |  |  |  |  |  |  |  |  |
| Widowed. divorced or single | - | - | - | - | -1.8 | 0.9 | -2.0 | 0.04 |
| Married or cohabiting | - | - | - | - | Ref. | . | . | . |
| Education |  |  |  |  |  |  |  |  |
| Secondary or vocational school | - | - | - | - | -7.0 | -0.1 | -7.0 | 0.05 |
| Matriculation or college examination | - | - | - | - | -1.2 | 4.5 | -1.2 | 0.25 |
| University degree | - | - | - | - | . | . | . | . |
| Income |  |  |  |  |  |  |  |  |
| Lowest quartile | - | - | - | - | -5.9 | -2.5 | -5.9 | <0.001 |
| Second lowest quartile | - | - | - | - | -4.2 | -1.2 | -4.2 | <0.001 |
| 3rd lowest quartile | - | - | - | - | -3.0 | -0.4 | -3.0 | 0.01 |
| Highest quartile | - | - | - | - | . | . | . | . |
| Interaction Terms |  |  |  |  |  |  |  |  |
| Manual workers * Phase in rel. to retirement -3 | 0.9 | 4.0 | 0.2 | 0.83 | -1.0 | 4.1 | -0.2 | 0.8 |
| Manual workers * Phase in rel. to retirement -2 | 7.1 | 3.7 | 1.9 | 0.06 | 5.9 | 3.8 | 1.6 | 0.12 |
| Manual workers * Phase in rel. to retirement -1 | 6.4 | 3.5 | 1.8 | 0.07 | 5.4 | 3.6 | 1.5 | 0.13 |
| Manual workers * Phase in rel. to retirement 0 | 3.6 | 3.5 | 1.1 | 0.3 | 2.9 | 3.5 | 0.8 | 0.42 |
| Manual workers * Phase in rel. to retirement 1 | 3.1 | 3.6 | 0.9 | 0.4 | 2.1 | 3.6 | 0.6 | 0.57 |
| Manual workers * Phase in rel. to retirement 2 | Ref. | . | . | . | Ref. | . | . | . |
| Non-manual employees * Phase in rel. to retirement -3 | 4.5 | 2.6 | 1.7 | 0.09 | 4.4 | 2.7 | 1.6 | 0.1 |
| Non-manual employees * Phase in rel. to retirement -2 | 5.7 | 2.4 | 2.4 | 0.02 | 6.0 | 2.4 | 2.5 | 0.01 |
| Non-manual employees * Phase in rel. to retirement -1 | 4.0 | 2.3 | 1.8 | 0.08 | 4.3 | 2.3 | 1.9 | 0.06 |
| Non-manual employees * Phase in rel. to retirement 0 | 3.5 | 2.2 | 1.6 | 0.12 | 4.1 | 2.3 | 1.8 | 0.07 |
| Non-manual employees * Phase in rel. to retirement 1 | 1.4 | 2.3 | 0.6 | 0.54 | 2.2 | 2.3 | 1.0 | 0.34 |
| Non-manual employees * Phase in rel. to retirement 2 | Ref. | . | . | . | Ref. | . | . | . |
| Semi-professionals * Phase in rel. to retirement -3 | -0.2 | 3.3 | -0.1 | 0.95 | -0.6 | 3.3 | -0.2 | 0.85 |
| Semi-professionals * Phase in rel. to retirement -2 | 3.6 | 3.0 | 1.2 | 0.22 | 3.0 | 3.0 | 1.0 | 0.32 |
| Semi-professionals * Phase in rel. to retirement -1 | 1.5 | 2.7 | 0.5 | 0.59 | 1.3 | 2.7 | 0.5 | 0.65 |
| Semi-professionals * Phase in rel. to retirement 0 | 2.4 | 2.7 | 0.9 | 0.38 | 2.2 | 2.7 | 0.8 | 0.41 |
| Semi-professionals * Phase in rel. to retirement 1 | 0.2 | 2.8 | 0.1 | 0.95 | 0.2 | 2.8 | 0.1 | 0.94 |
| Semi-professionals * Phase in rel. to retirement 2 | Ref. | . | . | . | Ref. | . | . | . |
| Professionals * Phase in rel. to retirement -3 | Ref. | . | . | . | Ref. | . | . | . |
| Professionals * Phase in rel. to retirement -2 | Ref. | . | . | . | Ref. | . | . | . |
| Professionals * Phase in rel. to retirement -1 | Ref. | . | . | . | Ref. | . | . | . |
| Professionals * Phase in rel. to retirement 0 | Ref. | . | . | . | Ref. | . | . | . |
| Professionals * Phase in rel. to retirement 1 | Ref. | . | . | . | Ref. | . | . | . |
| Professionals * Phase in rel. to retirement 2 | Ref. | . | . | . | Ref. | . | . | . |

Ref. indicates reference category

Phase in rel. to retirement indicates phase in relation to retirement
